# Highly precise protein-protein interaction prediction based on consensus between template-based and *de novo *docking methods

**DOI:** 10.1186/1753-6561-7-S7-S6

**Published:** 2013-12-20

**Authors:** Masahito Ohue, Yuri Matsuzaki, Takehiro Shimoda, Takashi Ishida, Yutaka Akiyama

**Affiliations:** 1 Graduate School of Information Science and Engineering, Tokyo Institute of Technology, 2-12-1-W8-76 Ookayama, Meguro-ku, Tokyo 152-8550, Japan; 2 Education Academy of Computational Life Sciences, Tokyo Institute of Technology, 2-12-1 Ookayama, Meguro-ku, Tokyo 152-8550, Japan; 3 Research Fellow of the Japan Society for the Promotion of Science

## Abstract

**Background:**

Elucidation of protein-protein interaction (PPI) networks is important for understanding disease mechanisms and for drug discovery. Tertiary-structure-based *in silico *PPI prediction methods have been developed with two typical approaches: a method based on template matching with known protein structures and a method based on *de novo *protein docking. However, the template-based method has a narrow applicable range because of its use of template information, and the *de novo *docking based method does not have good prediction performance. In addition, both of these *in silico *prediction methods have insufficient precision, and require validation of the predicted PPIs by biological experiments, leading to considerable expenditure; therefore, PPI prediction methods with greater precision are needed.

**Results:**

We have proposed a new structure-based PPI prediction method by combining template-based prediction and *de novo *docking prediction. When we applied the method to the human apoptosis signaling pathway, we obtained a precision value of 0.333, which is higher than that achieved using conventional methods (0.231 for PRISM, a template-based method, and 0.145 for MEGADOCK, a non-template-based method), while maintaining an F-measure value (0.285) comparable to that obtained using conventional methods (0.296 for PRISM, and 0.220 for MEGADOCK).

**Conclusions:**

Our consensus method successfully predicted a PPI network with greater precision than conventional template/non-template methods, which may thus reduce the cost of validation by laboratory experiments for confirming novel PPIs from predicted PPIs. Therefore, our method may serve as an aid for promoting interactome analysis.

## Introduction

Elucidation of regulatory relationships among the tens of thousands of protein species that function in a human cell is crucial for understanding the mechanisms underlying diseases and for the development of medicines [1]. Predicting protein-protein interaction (PPI) networks at the genome scale is one of the main topics in systems biology.

The methods used for PPI network prediction include primary-structure-based searching [2,3], evolutionary information-based methods [4], and tertiary-structure-based methods [5-7]. Tertiary-structure-based methods are attracting attention because they provide predicted protein complex structures and because they do not depend on homologous proteins. Tertiary structural information also provides powerful features for recognition [8,9] and is therefore useful for predicting binding affinity [10] in protein-protein complexes.

There are two typical approaches for tertiary-structure-based PPI predictions: a method based on template matching with known protein structures and another method based on *de novo *protein docking. The template-based method is based on the hypothesis that known complex structures or interface architectures can be used to model the complex formed between two target proteins. The hypothesis is logical, and this method provides good prediction performance when complex structural information is available as a template; however, if the template structure information is not available, performance is poor. In addition, because the interface architecture is not always similar for similar interactions, the template-based method has a narrow applicable range. In contrast, the *de novo *docking based method has a wide applicable range because it uses only tertiary structural information. However, because the advantage provided by existing template information is not utilized, the prediction performance is poor.

Tuncbag *et al*. developed a template-based PPI prediction method called PRISM [5], which is based on information regarding the interaction surface of crystalline complex structures. PRISM has been applied for predicting PPIs in a human apoptosis pathway [11] and a p53-protein-related pathway [12], and has contributed to the understanding of the structural mechanisms underlying some types of signal transduction. Ohue *et al*. developed a PPI prediction method called MEGADOCK [6] and Wass *et al*. developed a method [13] based on protein-protein docking without interaction surface information. MEGADOCK has been applied for PPI prediction for a bacterial chemotaxis pathway [7,14] and has contributed to the identification of protein pairs that may interact.

However, the prediction results of both template-based and *de novo *docking-based methods in these studies contained many false-positive predictions. PRISM obtained a precision value of 0.231 when applied to a human apoptosis pathway that consisted of 57 proteins, which was higher than the precision obtained with random prediction (precision value of 0.086), and MEGADOCK obtained a precision value of 0.400 when applied to a bacterial chemotaxis pathway that consisted of 13 proteins, which was higher than the precision obtained with random prediction (precision value of 0.253). To identify new PPIs, the prediction results need to be validated using biological experiments. For this purpose, obtaining a low number of predicted interaction candidates with high reliability is more important than obtaining a high number of predictions with low reliability. Thus, this paper aims to improve the reliability of the method used to obtain PPI predictions.

In this study, we combined two different PPI prediction methods to improve the precision of PPI prediction. Because PRISM is a template-based method, its prediction accuracy depends on the template dataset prepared. Only PPIs whose interaction surface structures are conserved are expected to be predicted. In contrast, MEGADOCK is a non-template-based method (also called *de novo *prediction), which has the demerit of generating false-positives for the cases in which no similar structures are seen in known complex structure databases; thus, template-based method would be ruled out from the prediction. However, in situations where template structures are not present in databases, MEGADOCK can still predict PPIs. This qualitative difference between the two methods typically makes their output different. Thus, the combination of both prediction methods may improve prediction accuracy, as the intersection set (AND set) of both results may contain fewer false-positives; this improvement in precision would also contribute to improvement in the prediction reliability provided by the use of just one method.

Such an approach is called a "meta" approach. Meta approaches have already been used in the field of protein tertiary structure prediction [15], and critical experiments have demonstrated improved performance of meta predictors when compared with the individual methods used in the meta predictors. The meta approach has also provided favorable results in protein domain prediction [16] and the prediction of disordered regions in proteins [17]. We have therefore proposed a new PPI prediction method based on the consensus between template-based and *de novo *docking methods. Generally, a meta prediction method may have low applicability because meta approaches require applicable conditions for every method in the approach. However, if structural information is available, the *de novo *docking method introduced in this study is always applicable with or without template information. Thus, the applicability of the consensus method is not narrower than that of a template-based method.

## Materials and methods

### Template-based PPI prediction

We used PRISM for template-based PPI prediction. PRISM uses two input datasets: the template set and the target set. The template set consists of interfaces extracted from protein pairs that are known to interact. The target set consists of protein chains whose interactions need to be predicted. The two sides of a template interface are compared with the surfaces of two target monomers by structural alignment. If regions of the target surfaces are similar to the complementary sides of the template interface, then these two targets are predicted to interact with each other through the template interface architecture.

The prediction algorithm consists of four steps: (1) interacting surface residues of target chains are extracted using Naccess [18]; (2) complementary chains of template interfaces are separated and structurally compared with each of the target surfaces by using MultiProt [19]; (3) the structural alignment results are filtered according to threshold values, and the resulting set of target surfaces is transformed onto the corresponding template interfaces to form a complex; and (4) the FiberDock [20] algorithm is used to refine the interactions to introduce flexibility, resolve steric clashes of side chains, compute the global energy of the complex, and rank the solutions according to their energies. When the computed energy of a protein pair is less than −10 kcal/mol, the pair is determined to "interact" (personal communication with Ms. Saliha Ece Acuner Ozbabacan, July 12, 2013). This prediction protocol has been described in detail in a previous study [5,11].

### PPI prediction based on the *de novo *docking method

For *de novo *protein docking-based PPI prediction, we used MEGADOCK version 2.6.2 [7]. MEGADOCK does not require template structures for prediction. The PPI prediction scheme used in this study consists of two steps. First, we conducted rigid-body docking calculations based on a simplified energy function considering shape complementarity, electrostatics, and hydrophobic interactions for all possible binary combinations of proteins in the target set. Using this process, we obtained a group of high-scoring docking complexes for each pair of proteins. Next, we applied ZRANK [21] to the predicted complex structures for more advanced binding energy calculation and re-ranked the docking results based on ZRANK energy scores. The deviation of the selected docking scores from the score distribution of high-ranked complexes was determined as a standardized score (Z-score) and was used to assess possible interactions. This prediction protocol has been described in previous studies [22,23]. Potential complexes that had no other high-scoring interactions nearby were rejected using structural differences. Thus, we considered likely binding pairs that had at least one populated area of high-scoring structures, one of which may be the true binding site.

### Consensus prediction method

In this study, we proposed a new meta-prediction method by evaluating the consensus between both previously used prediction methods. The proposed method consists of two steps: (1) prediction from the same target set by PRISM and MEGADOCK and (2) consideration that the method provides a prediction regarding target protein pair interaction only when both PRISM and MEGADOCK predict that the target protein pair interacts. Although some true-positives may be dropped by this method, the remaining predicted pairs are expected to have higher reliability because of the consensus between two prediction methods that have different characteristics.

### Dataset

In this study, we focused on the human apoptosis signaling pathway previously analyzed by PRISM because our prediction results can thus be compared directly to the results of the previous study. PRISM and MEGADOCK are based on three-dimensional protein structures and therefore can only be applied to proteins whose tertiary structures are available. Therefore, we searched among proteins involved in the human apoptosis pathway that were present in the Protein Data Bank (PDB) (accessed on July 28, 2012). We selected several proteins that had the highest resolution for the structural group that had high sequence similarity (>0.9) with the other proteins in the dataset [11]. After filtering according to resolution and sequence similarity, we obtained 158 PDB structures that corresponded to 57 proteins in the human apoptosis pathway described in KEGG (KEGG pathway ID: *hsa04210*) [24]. The PDB IDs in this structure dataset were the same as those used by Ozbabacan *et al. *[11]. Table 1 shows the list of PDB IDs and chains of this dataset.

**Table 1:** Protein and PDB ID list of human apoptosis pathway dataset

Protein Name	PDB ID (_Chain)					
AIF	1M6I_A					
AKT1	1UNQ_A	3CQW_A	3O96_A			
AKT2	1MRV_A	1O6K_A	1O6L_A	1P6S_A		
AKT3	2X18_A					
APAF1	1CY5_A	1Z6T_A	2YGS_A	3IZA_A	3YGS_C	
BCL-2	2W3L_A	2XA0_A				
BCL-XL	2B48_A	3FDL_A				
BID	2BID_A	2KBW_B				
Bax	1F16_A	2G5B_I	2XA0_C	3PK1_B		
CASP3	1RHQ_A	1RHQ_B	2DKO_A	2DKO_B	2J32_A	
CASP6	2WDP_A					
CASP7	1F1J_A	1I4O_A	1I51_A	1I51_B	2QL9_A	2QL9_B
CASP8	1QTN_A	1QTN_B	2FUN_B	3H11_B		
CASP9	1JXQ_A	1NW9_B	3D9T_C	3YGS_P		
Calpain1	1ZCM_A					
Calpain2	1KFU_L	2NQA_A				
Cn(CHP)	2E30_A					
Cn(CHP2)	2BEC_A					
Cn(PPP3CA)	1AUI_A	1MF8_A	2R28_C	3LL8_A		
Cn(PPP3R1)	1AUI_B	1MF8_B	3LL8_B			
CytC	1J3S_A					
DFF40	1IBX_A					
DFF45	1IBX_B	1IYR_A				
FADD	1A1W_A	2GF5_A	3EZQ_B			
FLIP	3H11_A					
Fas	3EWT_E	3EZQ_A				
IAP(BIRC2)	3D9T_A	3M1D_A	3MUP_A			
IAP(BIRC3)	2UVL_A	3EB5_A	3EB6_A	3M0A_D	3M0D_D	
IAP(BIRC4)	1G73_C	1I4O_C	1I51_E	1NW9_A	2ECG_A	2KNA_A
	2POI_A	3CM7_C				
IκBα	1IKN_D	1NFI_E				
IKK	2JVX_A	3BRT_B	3BRV_B	3CL3_D	3FX0_A	
IL-1(A)	2ILA_A					
IL-1(B)	1ITB_A	2NVH_A	3O4O_A			
IL-1R(1)	1ITB_B					
IL-1R(RAP)	3O4O_B					
IL-3	1JLI_A					
IL-3R	1EGJ_A					
IRAK2	3MOP_K					
IRAK4	2NRU_A	3MOP_G				
MyD88	2JS7_A	3MOP_A				
NF-κB(NFKB1)	1IKN_C	1NFI_B	1SVC_P	2DBF_A		
NF-κB(RELA)	1IKN_A	1NFI_A				
NGF	1WWW_V	2IFG_E				
PI3K(PIK3CA)	2ENQ_A	2V1Y_A	3HHM_A			
PI3K(PIK3CG)	1E8Y_A					
PI3K(PIK3R1)	1A0N_A	1H9O_A	1PBW_A	2IUG_A	2V1Y_B	3HHM_B
	3I5R_A					
PI3K(PIK3R2)	2KT1_A	2XS6_A	3MTT_A			
PRKACA	3AGM_A					
PRKAR2A	2IZX_A					
TNFα	1A8M_A	4TSV_A				
TNF-R1	1EXT_A	1ICH_A				
TP53	1AIE_A	1OLG_A	1XQH_B	1YC5_B	2B3G_B	2FOO_B
	2GS0_B	2K8F_B	2VUK_A	3D06_A	3DAB_B	3LW1_P
TRADD	1F3V_A					
TRAF2	1CZZ_A	1D00_A	1F3V_B	3KNV_A	3M0A_A	3M0D_A
TRAIL	1D4V_B	1DG6_A	1DU3_D			
TRAIL-R	1D4V_A	1DU3_A				
TrkA	1HE7_A	1SHC_B	1WWW_X	2IFG_A		

The abbreviations used are: AIF, apoptosis-inducing factor, mitochondrion-associated, 1 (AIFM1); AKT1, RAC-alpha serine/threonine-protein kinase; AKT2, RAC-beta serine/threonine-protein kinase; AKT3, RAC-gamma serine/threonine-protein kinase; APAF1, apoptotic peptidase activating factor 1; BCL-2, B-cell lymphoma 2; BCL-XL, BCL extra-large; BID, BH3 interacting domain death agonist; Bax, BCL-2-associated × protein; CASP3/6/7/8/9, caspase-3/6/7/8/9; Cn(CHP), calcineurin B homologous protein 1; Cn(CHP2), calcineurin B homologous protein 2; Cn(PPP3CA), protein phosphatase 3 catalytic subunit alpha isoform; Cn(PPP3R1), protein phosphatase 3 regulatory subunit 1; CytC, cytochrome C; DFF40, DNA fragmentation factor, 40kDa, beta polypeptide; DFF45, DNA fragmentation factor, 45kDa, alpha polypeptide; FADD, Fas-associated via death domain; FLIP, FLICE/CASP8 inhibitory protein (CASP8 and FADD-like apoptosis regulator, CFLAR); Fas, tumor necrosis factor receptor (TNF) superfamily member 6; IAP, inhibitor of apoptosis; BIRC2/3/4, baculoviral IAP repeat-containing protein 2/3/4; IκBα, nuclear factor of kappa light polypeptide gene enhancer in B-cells inhibitor alpha; IKK, inhibitor of nuclear factor kappa-B kinase; IL-1(A), interleukin-1 alpha; IL-1(B), interleukin-1 beta; IL-1R(1), type 1 interleukin-1 receptor; IL-1R(RAP), interleukin-1 receptor accessory protein; IL-3, interleukin-3; IL-3R, interleukin-3 receptor; IRAK2/4, interleukin-1 receptor-associated kinase 2/4; MyD88, myeloid differentiation primary response protein MyD88; NF-κB(NFKB1), nuclear factor of kappa light polypeptide gene enhancer in B-cells; NF-κB(RELA), nuclear factor of kappa light polypeptide gene enhancer in B-cells 3; NGF, nerve growth factor (beta polypeptide); PI3K, phosphatidylinositide 3-kinase; PIK3CA, PI3K subunit alpha; PIK3CG, PI3K subunit gamma; PIK3R1, PI3K regulatory subunit alpha; PIK3R2, PI3K regulatory subunit beta; PRKACA, cyclic adenosine monophosphate (cAMP)-dependent protein kinase catalytic subunit alpha; PRKAR2A, cAMP-dependent protein kinase type II-alpha regulatory subunit; TNFα, tumor necrosis factor; TNF-R1, TNF receptor superfamily member 1A; TP53, cellular tumor antigen p53; TRADD, TNF receptor type 1-associated death domain protein; TRAF2, TNF receptor-associated factor 2; TRAIL, TNF receptor superfamily member 10; TRAIL-R, TNF receptor superfamily member 10B; TrkA, neurotrophic tyrosine kinase receptor type 1.

Known PPIs were collected from the STRING database [25]. We used only experimental data in the literature obtained from STRING with a confidence score >0.5. The number of known PPIs was 137. Because the database does not contain existing self-interactions, we did not predict self-interactions. Thus, the number of target pairs was _57_**C**_2 _= 1,596.

### Evaluation of prediction performance

Here, we have defined #TP, #FP, #FN, #TN, precision, recall, and the F-measure, which we used to evaluate the prediction results: #TP is the number of predicted PPIs that were also found in STRING (true-positive), #FP is the number of predicted PPIs that were not in STRING (false-positive), #FN is the number of PPIs not predicted by the system even though the pair was found to interact in STRING (false-negative), and #TN is the number of negative predictions that were also not found in STRING (true-negative). Precision, recall, and the F-measure are represented as follows:

$$\text{precision}\;=\frac{\#\text{TP}}{\#\text{TP}+\#\text{FP}},\;\text{recall}\;=\frac{\#\text{TP}}{\#\text{TP}+\#\text{FN}},\;\text{F-measure}=\frac{2\cdot \#\text{TP}}{2\cdot \#\text{TP}+\#\text{FP}+\#\text{FN}},$$

where the F-measure is the harmonic mean of precision and recall. To identify new PPIs in biological experiments after *in silico *screening, precision is more important than recall to reduce the cost of validation.

## Results and Discussion

### Comparison of template- and non-template-based methods

**Figure 1:**
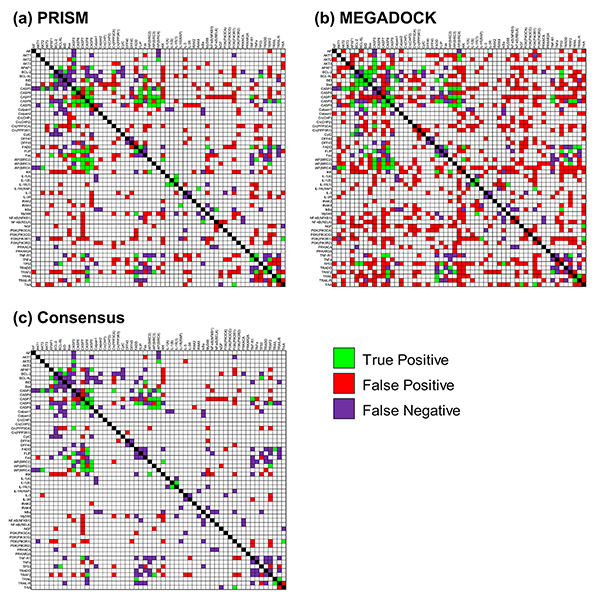
**Apoptosis prediction by the (a) PRISM, (b) MEGADOCK, and (c) consensus methods**. The green cells are true-positives, the red cells are false-positives, and the purple cells are false-negatives. The diagonal cells (black cells) have no PPI information in the STRING database and are excluded from the prediction targets.

Figure 1(a) and 1(b) show the prediction results for PRISM and MEGADOCK, respectively, as applied to a human apoptosis pathway. The threshold used for MEGADOCK prediction yielded the best value of the F-measure for this dataset. The diagonal line (black cells) in Figure 1 indicates self-interactions that were not considered as prediction targets. As shown in Figure 1, PRISM was performed with fewer FPs than MEGADOCK. Table 2 shows the evaluation of prediction results. With MEGADOCK, we obtained a lower value of precision and a higher value of recall relative to PRISM. When the F-measure was evaluated as a measure of overall performance, MEGADOCK showed lower values than PRISM. Predictions by MEGADOCK contained more FPs because, in contrast to PRISM, MEGADOCK does not restrict interface structures to those found in template structures. In contrast, PRISM obtained lower recall values than MEGADOCK because it only searched interactions whose interface structures could be found in the template set.

**Table 2:** Accuracy of human apoptosis pathway prediction

Method	#TP	#FP	#FN	#TN	Precision	Recall	F-measure
Consensus(AND)	34	68	103	1,391	0.333	0.248	0.285
OR	84	483	53	976	0.148	0.613	0.239
PRISM	56	186	81	1,273	0.231	0.409	0.296
MEGADOCK	62	365	75	1,094	0.145	0.453	0.220

### Results of the consensus prediction

**Figure 2:**
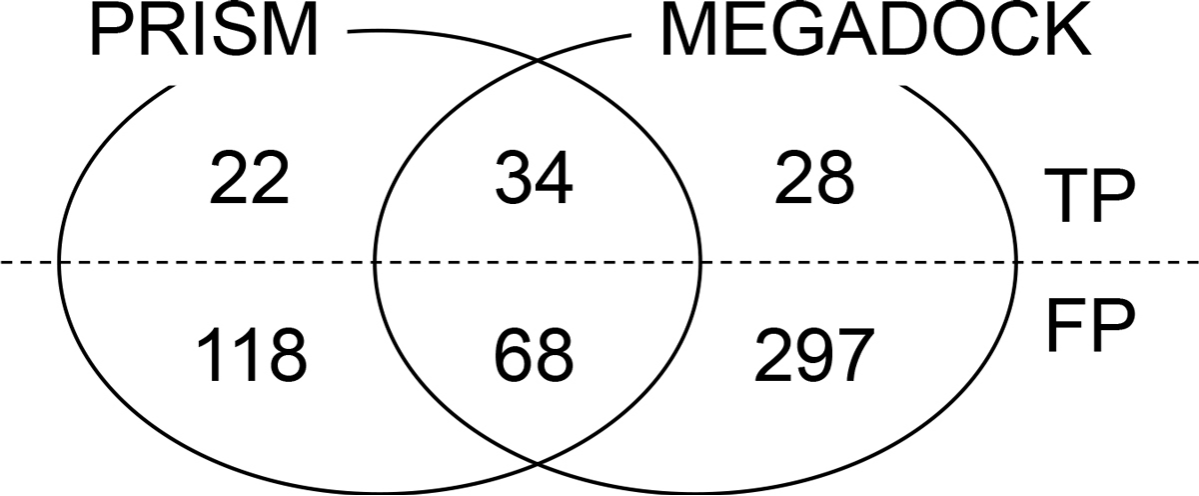
**Venn diagram of apoptosis pathway prediction results**. The common set (#TP = 34, #FP = 68) is denoted as "Consensus".

Figure 2 shows the Venn diagram of the number of TPs and FPs of the results of PRISM and MEGADOCK. A large difference was observed in the results obtained by the two methods. Thus, combining the prediction results of PRISM and MEGADOCK may provide better performance in PPI prediction. All of the predicted pairs of TPs and FPs are shown in Table S1 in Additional File 1.

Figure 1(c) shows the prediction obtained on consensus between PRISM (a) and MEGADOCK (b); notably, the number of FP samples greatly decreased. The first row of Table 2 shows that the consensus method obtained an F-measure value of 0.285, which was comparable to the PRISM result (F-measure = 0.296). The consensus prediction indicated a higher value of precision for the consensus method (0.333) than for PRISM (0.231). The consensus method yielded the highest precision value in the method shown in Table 2. This method is useful when validating unknown PPI predictions using biological experiments. In contrast, OR prediction demonstrated high recall (Table 2). Thus, the OR method will be useful when prediction with high sensitivity, e.g., in the initial construction of the draft PPI network from the relevant proteins, is required.

### An example of a false-positive pair and its predicted complex structure

The caspase-3 and caspase-7 pair is shown as an example of FP predictions in both PRISM and MEGADOCK with a particularly high evaluation value. Both caspase-3 and caspase-7 are effector caspases, which belong to a family of cysteine proteases that play essential roles in apoptosis. Effector caspases are activated by initiator caspases (e.g., caspase-2, 8, and 9), and then induce apoptotic cell death. Although the initiator and effector caspase cascade is well known, interactions among effector caspases are disputed [26].

**Figure 3:**
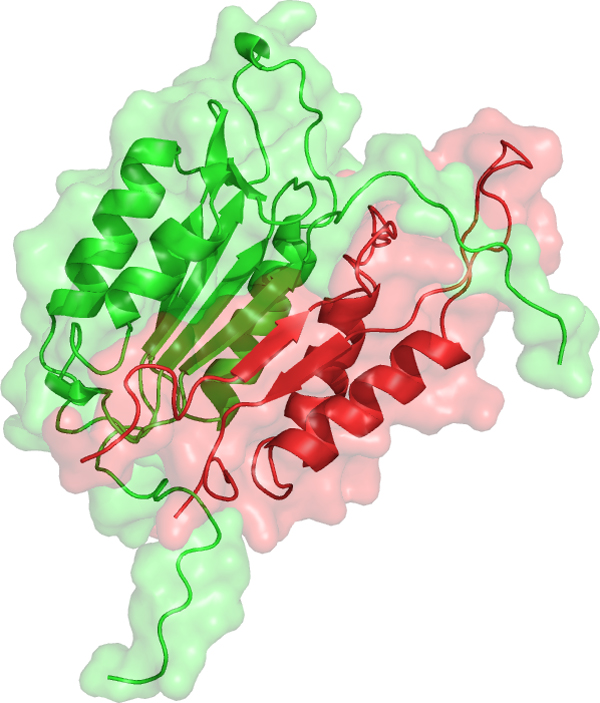
**Predicted complex structure of caspase-3 and caspase-7**. The red colored chain is caspase-3 protein (p17 subunit, PDB ID: 2QL9, chain B) and the green colored chain is caspase-7 (p10 subunit, PDB ID: 2DKO, chain A). The complex structure is predicted by MEGADOCK with the highest rank. This image was produced using PyMOL software [27].

The interaction of caspase-3 and caspase-7 was predicted with a high affinity score; the PRISM energy value was less than −190 kcal/mol and the MEGADOCK docking score was higher than 10,000. These values indicate a powerful affinity interaction. Figure 3 shows the predicted complex structure for caspase-3 and caspase-7. The predicted complex consists of 2DKO chain A (caspase-3, p17 subunit) and 2QL9 chain B (caspase-7, p10 subunit).

Additionally, 2DKO chain B (caspase-3, p12 subunit) and 2QL9 chain B, and 2QL9 chain A (caspase-7, p20 subunit) and 2DKO chain A, respectively, have similar structures. Thus, the predicted complex with each subunit swapped, as shown in Figure 3, is similar to the original heterodimer and possibly predicted to occur with a high score. The interaction among effector caspases, as in this case, has not been examined by biological experiments. In fact, another PPI prediction tool based on template structure and database information, PrePPI [28,29] (version 1.2.0), predicted the pair of caspase-3 and caspase-7 with a high score (the final probability value was 0.99). This situation is difficult to avoid in large-scale prediction problems. However, efforts such as the Negatome project [30] will help to improve this difficulty in the future.

### Relationship between the number of predicted positives and the number of structures

**Figure 4:**
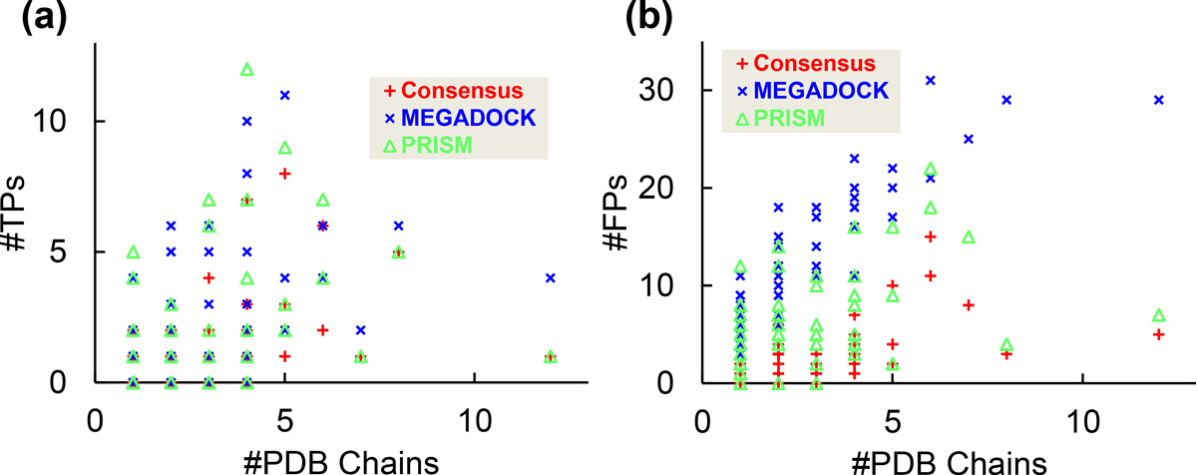
**Number of PDB chains vs. positive predictions**. (a) Shows the number of true-positives and (b) shows the number of false-positives. The horizontal axis is the number of PDB chains used in the interaction prediction, and the vertical axis is the number of positives predicted by using protein structures.

The structure-based PPI prediction method may generate positives with some bias regarding the type of proteins (rows and columns of Figure 1). From Table 1 and Figure 1, predictions with a large number of protein structures tend to generate more positive pairs. To verify this tendency, the number of PDB chain structures used for PPI prediction and the number of positive predicted pairs containing its protein are plotted in Figure 4. The #TPs are shown in Figure 4(a) and the #FPs are shown in Figure 4(b). Pearson's correlation coefficient *R *and the *P*-value for the correlation coefficient *t*-test are shown in Table 3.

**Table 3:** Correlation coefficient *R *and *P*-value of correlation test on Figure 4

Method	(a) #TPs		(b) #FPs	
	*R*	*P*-value	*R*	*P*-value
Consensus	0.477	1.784 × 10^-4^	0.594	1.121 × 10^-6^
PRISM	0.342	9.259 × 10^-3^	0.415	1.316 × 10^-3^
MEGADOCK	0.488	1.167 × 10^-4^	0.864	4.602 × 10^-18^

From the results of the *t*-tests, the number of chains and the number of positive predictions were clearly correlated with *P *< 0.05 in all cases, which suggests that the structure-based PPI prediction method should address the number of used protein structures without bias. For example, in a template matching-based method such as PRISM, a protein pair with more conformations of structures will have more matches in template complexes and a higher possibility of predicted interaction. In Table 3, the correlation coefficient values are particularly high in FP predictions. Therefore, for more precise prediction, we should consider one of the two ways: (i) how to generate the target set without multiple conformations in each protein and (ii) develop a correction method when the target set contains multiple conformations.

### Performance evaluation with various sensitivity parameters

**Figure 5:**
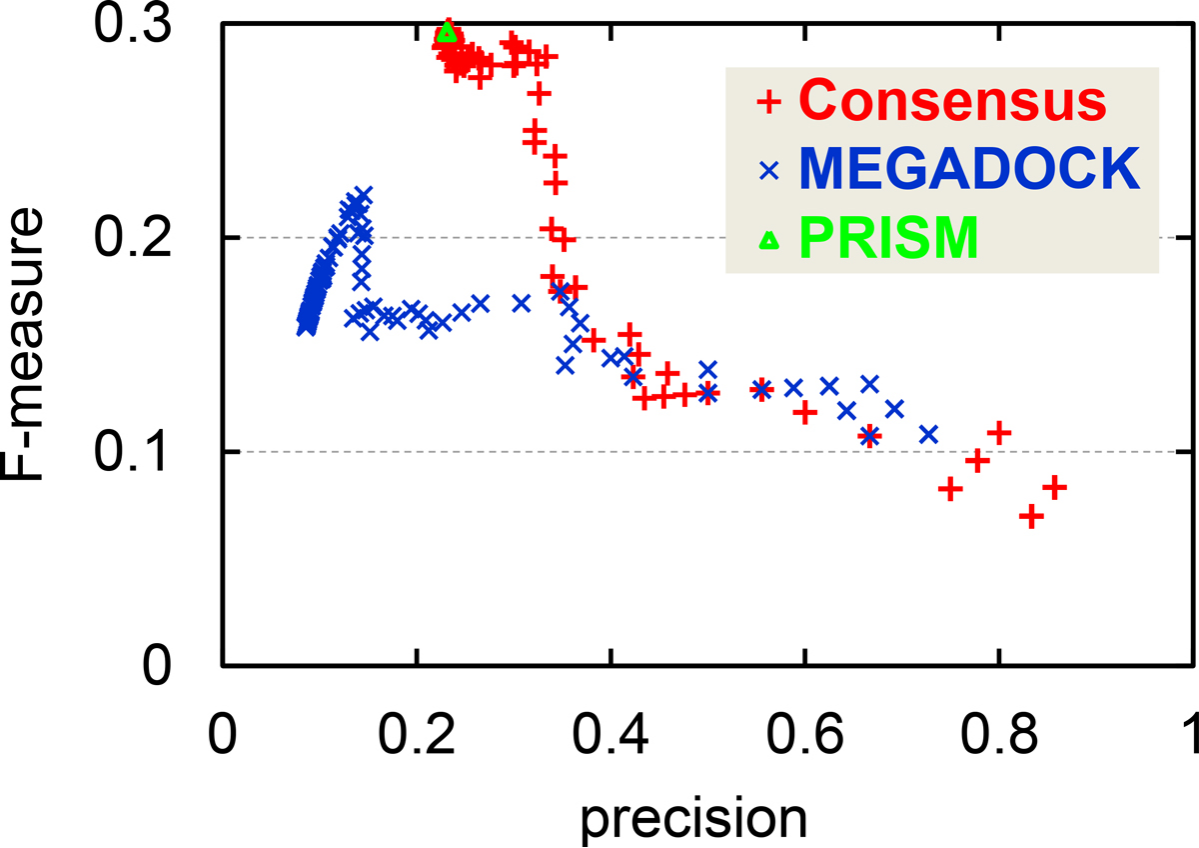
**F-measure vs. precision for predictions when the MEGADOCK threshold parameter is changed in the apoptosis pathway prediction**. The green triangle indicates the results of the PRISM prediction (Table 2).

In this study, we used a fixed threshold value for MEGADOCK that provided the best F-measure value for the target dataset. Figure 5 shows a plot of precision vs. F-measure value for prediction results with various threshold values for MEGADOCK. Figure 5 also plots the performance of the consensus method with various threshold values for MEGADOCK prediction while the threshold value for PRISM prediction was fixed. When the threshold value was changed in MEGADOCK, the plotted values remained in the region of low precision (0.0-0.2), and lower F-measure values were observed in the region of higher precision because of the decreased recall value. The consensus prediction method maintained a stable F-measure value when the value of precision was approximately 0.2-0.3, although the performance in the high-precision region (> 0.4) was inferior to that of MEGADOCK. In this region, the consensus prediction provides a better precision value than PRISM while maintaining the same F-measure value. Figure 5 clearly shows that the performance obtained by using the consensus method is better over a wide range of threshold values than the prediction obtained using only MEGADOCK.

**Figure 6:**
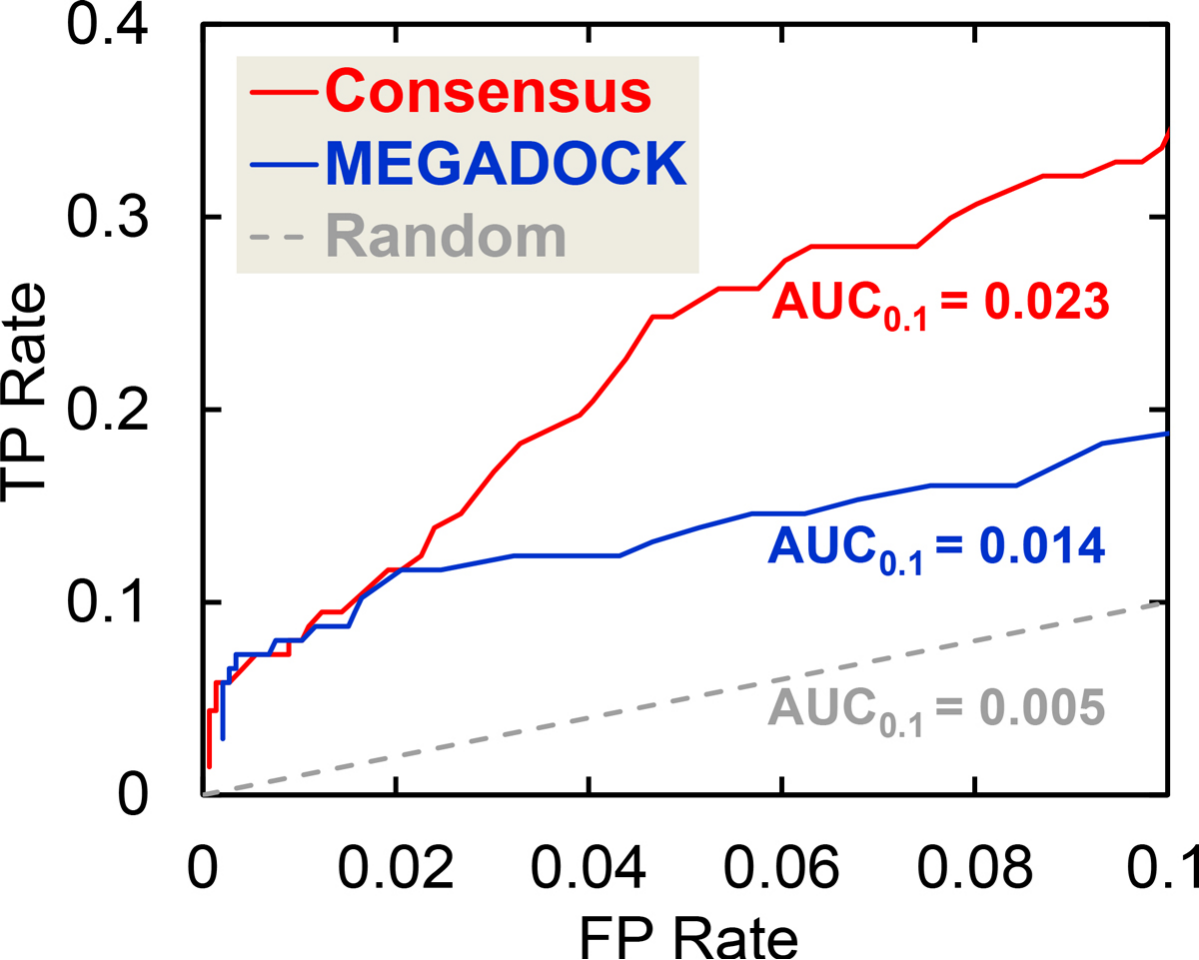
**ROC_0.1 _curves obtained when the MEGADOCK threshold parameter is changed in the apoptosis pathway prediction**. AUC_0.1 _is the area under the ROC_0.1 _curve. For the 0-0.1 FP rate range here, a random prediction produced an AUC_0.1 _of 0.005.

The AUC, i.e., the area under the ROC curve [31], is a more general and effective statistical measure. The ROC_0.1 _curves, which include the ROC curves up to an FP rate of 0.1, are shown in Figure 6. ROC curves were created by plotting the TP rate (#TP/(#TP+#FN)) against the FP rate (#FP/(#FP+#TN)). Regions with high FP rates are not useful for prediction because many FPs are generated, e.g., an FP rate of 0.2 represents #FP = 292. The ROC_0.1 _curve was thus considered to favor methods that produce a high TP rate at low FP rates, and the associated area under the curve is referred to as AUC_0.1_. A perfect prediction will produce an AUC_0.1 _of (0.1 × 1 =) 0.1, whereas a random prediction will result in an AUC_0.1 _of (0.1 × 0.1/2 =) 0.005. Figure 6 shows that the consensus prediction (AUC_0.1 _= 0.023) is better than the MEGADOCK (AUC_0.1 _= 0.014) and random predictions (AUC_0.1 _= 0.005).

## Conclusions

In this study, we propose a new PPI network prediction method based on the consensus between template-based prediction and non-template-based prediction. The consensus method successfully predicted the PPI network more accurately than the conventional single template/non-template method. Because such precise prediction can reduce biological screening costs, it will promote interactome analysis. For further improvement of prediction performance, it is necessary to further improve the combination of the two techniques, e.g., by using a strategy other than taking a simple AND/OR consensus. For example, biological information such as biochemical function and subcellular localization information could be used.

## List of abbreviations

PPI: protein-protein interaction; PDB: protein data bank; KEGG: Kyoto encyclopedia of genes and genomes; TP: true-positive; FP: false-positive; FN: false-negative; TN: true-negative; ROC: receiver operating characteristic; AUC: area under the (ROC) curve.

## Competing interests

The authors declare that they have no competing interests.

## Authors' contributions

MO developed the consensus interaction prediction method, designed the human apoptosis pathway problem, and wrote the manuscript. MO and YM performed the computational experiments and validated the results. TS performed the PRISM experiments. TI assisted with the method design. YA supervised and directed the entire study. All authors read and approved the final manuscript.

## Supplementary Material

Additional file 1 **Supplementary table for predicted list**. Table S1: The list of all true-positive pairs and false-positive pairs predicted by the PRISM, MEGADOCK, and consensus methods; (a) the true-positive list of PRISM predictions, (b) the false-positive list of PRISM predictions, (c) the true-positive list of MEGADOCK predictions, (d) the false-positive list of MEGADOCK predictions, (e) the true-positive list of consensus predictions, and (f) the false-positive list of consensus predictions. Click here for file

## Declarations

The publication fee of this article was funded by Tokyo Institute of Technology. This work was supported in part by a Grant-in-Aid for JSPS Fellows (238750), a Grant-in-Aid for Research and Development of The Next-Generation Integrated Simulation of Living Matter (ISLiM), and by the Education Academy of Computational Life Sciences (ACLS) at the Tokyo Institute of Technology, all of which were from the Ministry of Education, Culture, Sports, Science, and Technology of Japan (MEXT).

This article has been published as part of *BMC Proceedings *Volume 7 Supplement 7, 2013: Proceedings of the Great Lakes Bioinformatics Conference 2013. The full contents of the supplement are available online at http://www.biomedcentral.com/bmcproc/supplements/7/S7.
