# Supplementary material for: Highly precise protein-protein interaction prediction based on consensus between template-based and de novo docking methods
Source: BMC Proc. 2013 Dec 20;7(Suppl 7):S6. doi: 10.1186/1753-6561-7-S7-S6 (PMC4044902; doi:10.1186/1753-6561-7-S7-S6)
Supplement: Additional file 1 — Supplementary table for predicted list. Table S1: The list of all true-positive pairs and false-positive pairs predicted by the PRISM, MEGADOCK, and consensus methods; (a) the true-positive list of PRISM predictions, (b) the false-positive list of PRISM predictions, (c) the true-positive list of MEGADOCK predictions, (d) the false-positive list of MEGADOCK predictions, (e) the true-positive list of consensus predictions, and (f) the false-positive list of consensus predictions. [file 1753-6561-7-S7-S6-S1.PDF]

Supplementary materials for

Highly precise protein-protein interaction prediction based on consensus between template-based and *de novo* docking methods

Masahito Ohue, Yuri Matsuzaki, Takehiro Shimoda, Takashi Ishida and Yutaka Akiyama
Contact: akiyama@cs.titech.ac.jp

Table S1: The list of all true-positive pairs and false-positive pairs predicted by PRISM, MEGADOCK and Consensus method.

(a) The true-positive list of PRISM predictions

AKT2 - CASP3	CASP6 - FLIP	CASP9 - IAP(BIRC4)
AKT2 - IAP(BIRC4)	CASP6 - IAP(BIRC2)	FADD - FLIP
APAF1 - BCL-XL	CASP7 - CASP8	FADD - MyD88
APAF1 - CASP3	CASP7 - CASP9	FADD - TNF-R1
APAF1 - CASP9	CASP7 - FLIP	FADD - TRADD
APAF1 - Fas	CASP7 - IAP(BIRC2)	FADD - TRAIL
BCL-2 - BID	CASP7 - IAP(BIRC3)	IAP(BIRC2) - IKK
BCL-2 - Cn(PPP3CA)	CASP7 - IAP(BIRC4)	IAP(BIRC2) - TNFα
BCL-XL - TP53	CASP7 - TNF-R1	IAP(BIRC3) - TRAF2
BID - CASP8	CASP8 - CASP9	IL-1(A) - IL-1R(1)
BID - Fas	CASP8 - FADD	IL-1(B) - IL-1R(1)
CASP3 - CASP6	CASP8 - FLIP	IL-1R(RAP) - IRAK4
CASP3 - CASP8	CASP8 - IAP(BIRC2)	IκBα - NF-κB(RELA)
CASP3 - CASP9	CASP8 - IAP(BIRC4)	PI3K(PIK3R1) - TrkA
CASP3 - Cn(PPP3CA)	CASP8 - IKK	TNF-R1 - TNFα
CASP3 - FLIP	CASP8 - TRAF2	TNFα - TRAF2
CASP3 - IAP(BIRC2)	CASP8 - TRAIL-R	TRADD - TRAF2
CASP3 - IAP(BIRC4)	CASP9 - IAP(BIRC2)	TRAIL - TRAIL-R
CASP6 - CASP8	CASP9 - IAP(BIRC3)	

(b) The false-positive list of PRISM predictions

AIF - TRAF2	CASP6 - DFF45	DFF45 - TRAF2
AKT1 - Fas	CASP6 - IKK	FADD - PI3K(PIK3CA)
AKT1 - TrkA	CASP6 - PI3K(PIK3R1)	FADD - PI3K(PIK3CG)
AKT2 - Bax	CASP6 - TRAF2	FADD - PI3K(PIK3R1)
AKT2 - FADD	CASP6 - TRAIL-R	FADD - TNFα
AKT2 - IL-3	CASP7 - CytC	FLIP - TrkA
AKT3 - Cn(PPP3CA)	CASP7 - FADD	Fas - IKK
AKT3 - Cn(PPP3R1)	CASP7 - Fas	Fas - MyD88
AKT3 - MyD88	CASP7 - IKK	Fas - NF-κB(NFKB1)
AKT3 - TrkA	CASP7 - IRAK4	Fas - NF-κB(RELA)
APAF1 - Bax	CASP7 - MyD88	Fas - TP53
APAF1 - CASP7	CASP7 - NF-κB(NFKB1)	Fas - TRAF2
APAF1 - FADD	CASP7 - NF-κB(RELA)	IAP(BIRC2) - MyD88
APAF1 - IKK	CASP7 - NGF	IAP(BIRC2) - PI3K(PIK3R1)
APAF1 - IL-3	CASP7 - PI3K(PIK3CA)	IAP(BIRC3) - NGF
APAF1 - IRAK4	CASP7 - PI3K(PIK3CG)	IAP(BIRC4) - MyD88
APAF1 - PI3K(PIK3R1)	CASP7 - PI3K(PIK3R1)	IAP(BIRC4) - TRADD
APAF1 - TRAF2	CASP7 - TNFα	IKK - IRAK2
APAF1 - TrkA	CASP7 - TRAF2	IKK - MyD88
BCL-2 - FADD	CASP8 - Cn(PPP3CA)	IKK - NF-κB(NFKB1)
BCL-2 - IKK	CASP8 - IAP(BIRC3)	IKK - PI3K(PIK3R1)
BCL-2 - NGF	CASP8 - IL-1R(1)	IKK - PI3K(PIK3R2)
BCL-2 - PI3K(PIK3R1)	CASP8 - PI3K(PIK3R1)	IKK - TP53
BCL-2 - TRAF2	CASP9 - Calpain1	IKK - TRAF2
BCL-XL - Cn(PPP3CA)	CASP9 - DFF40	IL-1R(1) - IL-3

BCL-XL - Fas	CASP9 - FADD	IL-1R(1) - TNF-R1
BCL-XL - IKK	CASP9 - FLIP	IL-1R(1) - TNFα
BCL-XL - IL-1R(1)	CASP9 - IL-3	IL-1R(RAP) - TNFα
BCL-XL - IL-3	CASP9 - IL-3R	IL-3 - MyD88
BCL-XL - PI3K(PIK3R1)	CASP9 - IRAK2	IL-3 - PI3K(PIK3R1)
BCL-XL - TRAF2	CASP9 - IRAK4	IL-3 - TNF-R1
BCL-XL - TRAIL-R	CASP9 - TNFα	IL-3 - TRAF2
BID - CASP7	Calpain1 - FADD	IL-3 - TrkA
BID - Cn(PPP3CA)	Calpain1 - IAP(BIRC4)	IL-3R - TNF-R1
BID - IAP(BIRC2)	Calpain1 - PI3K(PIK3R2)	IL-3R - TNFα
BID - IKK	Calpain2 - IAP(BIRC2)	IL-3R - TRAF2
Bax - CASP9	Calpain2 - PI3K(PIK3R2)	IL-3R - TRAIL-R
Bax - Calpain2	Calpain2 - TRAF2	IRAK4 - TNF-R1
Bax - Cn(CHP)	Cn(PPP3CA) - DFF45	MyD88 - PI3K(PIK3R1)
Bax - Cn(CHP2)	Cn(PPP3CA) - Fas	MyD88 - PI3K(PIK3R2)
Bax - Cn(PPP3CA)	Cn(PPP3CA) - IAP(BIRC2)	NF-κB(NFKB1) - PI3K(PIK3R1)
Bax - Cn(PPP3R1)	Cn(PPP3CA) - IKK	NF-κB(RELA) - NGF
Bax - Fas	Cn(PPP3CA) - MyD88	NF-κB(RELA) - PI3K(PIK3CA)
Bax - IAP(BIRC4)	Cn(PPP3CA) - PI3K(PIK3R2)	NGF - TRAF2
Bax - IRAK2	Cn(PPP3CA) - PRKACA	NGF - TRAIL
Bax - MyD88	Cn(PPP3CA) - TNFα	PI3K(PIK3CA) - TNFα
Bax - PI3K(PIK3CA)	Cn(PPP3CA) - TP53	PI3K(PIK3CG) - TNFα
Bax - PI3K(PIK3CG)	Cn(PPP3CA) - TRAF2	PI3K(PIK3CG) - TRAF2
Bax - TNFα	Cn(PPP3R1) - DFF40	PI3K(PIK3CG) - TRAIL
Bax - TrkA	Cn(PPP3R1) - Fas	PI3K(PIK3R1) - TNF-R1
CASP3 - CASP7	Cn(PPP3R1) - IL-3	PI3K(PIK3R1) - TRAF2
CASP3 - Cn(CHP)	Cn(PPP3R1) - TNFα	PI3K(PIK3R2) - TNF-R1
CASP3 - Fas	CytC - PI3K(PIK3R2)	PI3K(PIK3R2) - TRADD
CASP3 - IKK	DFF40 - IL-3R	PI3K(PIK3R2) - TRAF2
CASP3 - IRAK4	DFF40 - MyD88	TNFα - TRAIL
CASP3 - NF-κB(NFKB1)	DFF40 - TNFα	TNFα - TRAIL-R
CASP3 - PI3K(PIK3R2)	DFF40 - TP53	TP53 - TRAF2
CASP3 - TNF-R1	DFF40 - TRAF2	TP53 - TRAIL
CASP3 - TRAF2	DFF45 - FADD	TP53 - TRAIL-R
CASP6 - CASP7	DFF45 - Fas	TRAF2 - TRAIL-R
CASP6 - CASP9	DFF45 - IL-3	TRAF2 - TrkA
CASP6 - Cn(PPP3CA)	DFF45 - PI3K(PIK3R1)	TRAIL-R - TrkA

(c) The true-positive list of MEGADOCK predictions

AKT1 - IAP(BIRC4)	BID - Fas	CASP9 - IAP(BIRC2)
AKT2 - CASP3	CASP3 - CASP8	CASP9 - IAP(BIRC3)
AKT2 - IAP(BIRC4)	CASP3 - CASP9	CASP9 - IAP(BIRC4)
AKT3 - CASP3	CASP3 - Cn(PPP3CA)	FADD - Fas
APAF1 - BCL-XL	CASP3 - FLIP	FADD - IKK
APAF1 - CASP3	CASP3 - IAP(BIRC2)	FADD - TRAIL
APAF1 - CASP8	CASP3 - IAP(BIRC3)	FADD - TRAIL-R
APAF1 - CASP9	CASP3 - IAP(BIRC4)	FLIP - TP53
APAF1 - CytC	CASP7 - CASP8	FLIP - TRAF2
BCL-2 - BID	CASP7 - CASP9	IAP(BIRC2) - IKK
BCL-2 - Bax	CASP7 - IAP(BIRC2)	IAP(BIRC2) - TRADD
BCL-2 - CASP3	CASP7 - IAP(BIRC3)	IAP(BIRC3) - TRAF2
BCL-2 - Cn(PPP3R1)	CASP7 - IAP(BIRC4)	IL-1(B) - IL-1R(1)
BCL-2 - TP53	CASP7 - TNF-R1	IL-1R(1) - MyD88
BCL-XL - BID	CASP8 - CASP9	IL-1R(RAP) - PI3K(PIK3R1)
BCL-XL - Bax	CASP8 - FADD	IκBα - TP53
BCL-XL - CASP9	CASP8 - FLIP	NGF - TrkA
BCL-XL - TP53	CASP8 - IAP(BIRC4)	PI3K(PIK3R1) - TrkA
BID - Bax	CASP8 - TNF-R1	TNF-R1 - TRAF2
BID - Calpain1	CASP8 - TRAF2	TRAIL - TRAIL-R
BID - FADD	CASP8 - TRAIL	

(d) The false-positive list of MEGADOCK predictions

AIF - AKT2	CASP3 - PI3K(PIK3R1)	FADD - TP53
AIF - BCL-XL	CASP3 - PI3K(PIK3R2)	FLIP - IAP(BIRC4)
AIF - CASP7	CASP3 - TNFα	FLIP - IKK
AIF - Calpain2	CASP3 - TP53	FLIP - IL-1R(RAP)
AIF - PI3K(PIK3R1)	CASP3 - TRAF2	FLIP - NGF
AIF - TP53	CASP3 - TRAIL	FLIP - PI3K(PIK3R2)
AIF - TRAIL	CASP3 - TRAIL-R	Fas - IAP(BIRC3)
AIF - TrkA	CASP3 - TrkA	Fas - PRKACA
AKT1 - APAF1	CASP6 - CASP7	Fas - TP53
AKT1 - BCL-2	CASP6 - IAP(BIRC3)	Fas - TRAIL
AKT1 - Bax	CASP6 - IAP(BIRC4)	IAP(BIRC2) - IL-1(A)
AKT1 - CASP8	CASP6 - IKK	IAP(BIRC2) - IL-3
AKT1 - DFF40	CASP6 - TRAIL	IAP(BIRC2) - IRAK2
AKT1 - DFF45	CASP7 - Calpain1	IAP(BIRC2) - NF-κB(NFKB1)
AKT1 - FADD	CASP7 - Calpain2	IAP(BIRC2) - NGF
AKT1 - Fas	CASP7 - Cn(CHP2)	IAP(BIRC2) - TRAIL-R
AKT1 - IAP(BIRC2)	CASP7 - Cn(PPP3R1)	IAP(BIRC3) - NF-κB(RELA)
AKT1 - IAP(BIRC3)	CASP7 - CytC	IAP(BIRC3) - NGF
AKT1 - IKK	CASP7 - FADD	IAP(BIRC3) - PI3K(PIK3R2)
AKT1 - IL-3R	CASP7 - Fas	IAP(BIRC3) - PRKACA
AKT1 - NF-κB(NFKB1)	CASP7 - IL-1(B)	IAP(BIRC3) - PRKAR2A
AKT1 - NF-κB(RELA)	CASP7 - IκBα	IAP(BIRC3) - TP53
AKT1 - PI3K(PIK3CG)	CASP7 - MyD88	IAP(BIRC3) - TRAIL-R
AKT1 - PI3K(PIK3R2)	CASP7 - NF-κB(NFKB1)	IAP(BIRC4) - IKK
AKT1 - TRAF2	CASP7 - NF-κB(RELA)	IAP(BIRC4) - IL-1(B)
AKT2 - APAF1	CASP7 - NGF	IAP(BIRC4) - IL-1R(1)
AKT2 - BCL-XL	CASP7 - PI3K(PIK3CA)	IAP(BIRC4) - IL-1R(RAP)
AKT2 - CASP9	CASP7 - PI3K(PIK3R1)	IAP(BIRC4) - IL-3R
AKT2 - Calpain2	CASP7 - PI3K(PIK3R2)	IAP(BIRC4) - IRAK2
AKT2 - Cn(CHP2)	CASP7 - PRKACA	IAP(BIRC4) - IκBα
AKT2 - FLIP	CASP7 - TNFα	IAP(BIRC4) - MyD88
AKT2 - IAP(BIRC3)	CASP7 - TP53	IAP(BIRC4) - NF-κB(NFKB1)
AKT2 - IL-1(B)	CASP7 - TRADD	IAP(BIRC4) - PI3K(PIK3CA)
AKT2 - IL-3	CASP7 - TRAF2	IAP(BIRC4) - PI3K(PIK3CG)
AKT2 - MyD88	CASP7 - TRAIL	IAP(BIRC4) - PI3K(PIK3R1)

AKT2 - NF-κB(RELA)	CASP7 - TrkA	IAP(BIRC4) - PI3K(PIK3R2)
AKT2 - PI3K(PIK3CA)	CASP8 - Cn(CHP2)	IAP(BIRC4) - TNF-R1
AKT2 - PI3K(PIK3R1)	CASP8 - Cn(PPP3CA)	IAP(BIRC4) - TP53
AKT2 - TP53	CASP8 - DFF45	IAP(BIRC4) - TRADD
AKT2 - TRAF2	CASP8 - IAP(BIRC3)	IAP(BIRC4) - TRAF2
AKT2 - TRAIL-R	CASP8 - MyD88	IAP(BIRC4) - TRAIL
AKT2 - TrkA	CASP8 - NGF	IAP(BIRC4) - TRAIL-R
AKT3 - APAF1	CASP8 - PI3K(PIK3R1)	IAP(BIRC4) - TrkA
AKT3 - CASP7	CASP8 - TP53	IKK - IL-3
AKT3 - CASP9	CASP8 - TrkA	IKK - IRAK4
AKT3 - FADD	CASP9 - Cn(PPP3R1)	IKK - MyD88
APAF1 - Bax	CASP9 - IL-1R(RAP)	IKK - NF-κB(NFKB1)
APAF1 - CASP6	CASP9 - IRAK2	IKK - NF-κB(RELA)
APAF1 - CASP7	CASP9 - IκBα	IKK - PI3K(PIK3R1)
APAF1 - IAP(BIRC3)	CASP9 - PI3K(PIK3R1)	IKK - TP53
APAF1 - IAP(BIRC4)	CASP9 - PI3K(PIK3R2)	IKK - TRADD
APAF1 - IKK	CASP9 - TNFα	IKK - TRAF2
APAF1 - IL-1R(RAP)	CASP9 - TRAF2	IKK - TRAIL
APAF1 - NF-κB(NFKB1)	CASP9 - TRAIL	IKK - TrkA
APAF1 - NF-κB(RELA)	Calpain1 - IAP(BIRC3)	IL-1(A) - IL-1R(RAP)
APAF1 - NGF	Calpain1 - NGF	IL-1(A) - PI3K(PIK3CA)
APAF1 - PI3K(PIK3CG)	Calpain2 - IAP(BIRC4)	IL-1(A) - PI3K(PIK3R1)
APAF1 - PI3K(PIK3R1)	Calpain2 - IKK	IL-1(A) - TP53
APAF1 - PRKAR2A	Calpain2 - IL-1(B)	IL-1(A) - TRAIL
APAF1 - TNFα	Calpain2 - NGF	IL-1(B) - IRAK4
APAF1 - TP53	Calpain2 - PRKACA	IL-1(B) - PI3K(PIK3CG)
APAF1 - TRAF2	Calpain2 - TP53	IL-1(B) - PI3K(PIK3R1)
BCL-2 - DFF45	Calpain2 - TRAIL-R	IL-1(B) - TP53
BCL-2 - IAP(BIRC4)	Calpain2 - TrkA	IL-1(B) - TRAIL-R
BCL-2 - IKK	Cn(CHP) - IKK	IL-1R(1) - IRAK2
BCL-2 - IL-1R(1)	Cn(CHP) - IL-1R(1)	IL-1R(1) - IRAK4
BCL-2 - NGF	Cn(CHP) - IRAK4	IL-1R(1) - IκBα
BCL-2 - PI3K(PIK3R1)	Cn(CHP) - NF-κB(NFKB1)	IL-1R(1) - PI3K(PIK3R1)
BCL-2 - TRAIL	Cn(CHP) - PI3K(PIK3CG)	IL-1R(RAP) - IκBα
BCL-2 - TRAIL-R	Cn(CHP2) - IAP(BIRC4)	IL-1R(RAP) - TP53
BCL-XL - CASP6	Cn(CHP2) - IL-1R(RAP)	IL-3 - NF-κB(NFKB1)
BCL-XL - CASP7	Cn(CHP2) - NF-κB(NFKB1)	IL-3 - PI3K(PIK3R2)
BCL-XL - Cn(CHP)	Cn(CHP2) - NGF	IL-3 - TNFα
BCL-XL - Cn(PPP3CA)	Cn(CHP2) - PI3K(PIK3CA)	IL-3 - TRAF2
BCL-XL - IAP(BIRC2)	Cn(CHP2) - PRKACA	IL-3R - NGF
BCL-XL - IAP(BIRC3)	Cn(PPP3CA) - Cn(PPP3R1)	IL-3R - TNF-R1
BCL-XL - IKK	Cn(PPP3CA) - FADD	IL-3R - TRAF2
BCL-XL - NF-κB(NFKB1)	Cn(PPP3CA) - IAP(BIRC2)	IL-3R - TrkA
BCL-XL - NGF	Cn(PPP3CA) - IAP(BIRC3)	IRAK2 - NF-κB(NFKB1)
BCL-XL - TRAF2	Cn(PPP3CA) - IL-1(A)	IRAK2 - TRAF2
BCL-XL - TRAIL	Cn(PPP3CA) - IL-1(B)	IRAK4 - PI3K(PIK3R2)
BCL-XL - TrkA	Cn(PPP3CA) - IL-1R(1)	IRAK4 - TP53
BID - CASP7	Cn(PPP3CA) - IRAK4	IRAK4 - TRAF2
BID - Cn(CHP2)	Cn(PPP3CA) - IκBα	IRAK4 - TrkA
BID - Cn(PPP3CA)	Cn(PPP3CA) - MyD88	IκBα - PI3K(PIK3CA)
BID - Cn(PPP3R1)	Cn(PPP3CA) - NGF	IκBα - TrkA
BID - IKK	Cn(PPP3CA) - PI3K(PIK3CA)	MyD88 - TP53
BID - IRAK4	Cn(PPP3CA) - PI3K(PIK3CG)	MyD88 - TrkA
BID - MyD88	Cn(PPP3CA) - TP53	NF-κB(NFKB1) - NF-κB(RELA)
BID - NF-kB(RELA)	Cn(PPP3CA) - TRAF2	NF-κB(NFKB1) - TNFα
BID - PI3K(PIK3CA)	Cn(PPP3CA) - TRAIL	NF-κB(RELA) - TNFα
BID - TP53	Cn(PPP3CA) - TrkA	NF-κB(RELA) - TP53
BID - TRAIL	Cn(PPP3R1) - CytC	NF-κB(RELA) - TRAF2
BID - TrkA	Cn(PPP3R1) - DFF40	NF-κB(RELA) - TRAIL

Bax - CASP7	Cn(PPP3R1) - IAP(BIRC4)	NF-κB(RELA) - TrkA
Bax - CASP8	Cn(PPP3R1) - IKK	NGF - PI3K(PIK3R1)
Bax - CytC	Cn(PPP3R1) - IL-1(B)	NGF - TP53
Bax - Fas	Cn(PPP3R1) - IκBα	PI3K(PIK3CA) - PI3K(PIK3CG)
Bax - IAP(BIRC2)	Cn(PPP3R1) - NF-κB(NFKB1)	PI3K(PIK3CA) - PI3K(PIK3R1)
Bax - IAP(BIRC3)	Cn(PPP3R1) - PI3K(PIK3CA)	PI3K(PIK3CA) - TP53
Bax - IAP(BIRC4)	Cn(PPP3R1) - TNF-R1	PI3K(PIK3CA) - TRAF2
Bax - IL-1R(RAP)	Cn(PPP3R1) - TP53	PI3K(PIK3CA) - TrkA
Bax - IL-3	Cn(PPP3R1) - TRAF2	PI3K(PIK3R1) - PI3K(PIK3R2)
Bax - MyD88	Cn(PPP3R1) - TrkA	PI3K(PIK3R1) - TNF-R1
Bax - NF-κB(RELA)	CytC - FADD	PI3K(PIK3R1) - TNFα
Bax - PI3K(PIK3R1)	CytC - TP53	PI3K(PIK3R1) - TP53
Bax - TNF-R1	DFF40 - IL-3	PI3K(PIK3R1) - TRADD
Bax - TP53	DFF40 - MyD88	PI3K(PIK3R1) - TRAF2
Bax - TRADD	DFF40 - PI3K(PIK3CA)	PI3K(PIK3R1) - TRAIL
Bax - TRAIL-R	DFF40 - PI3K(PIK3R1)	PI3K(PIK3R2) - TNF-R1
CASP3 - CASP7	DFF40 - TrkA	PI3K(PIK3R2) - TRAF2
CASP3 - Calpain2	DFF45 - IAP(BIRC2)	PI3K(PIK3R2) - TRAIL
CASP3 - Cn(CHP2)	DFF45 - NF-κB(NFKB1)	PI3K(PIK3R2) - TrkA
CASP3 - Cn(PPP3R1)	DFF45 - NGF	PRKACA - TRAIL-R
CASP3 - IL-1(A)	DFF45 - PI3K(PIK3CA)	TNFα - TP53
CASP3 - IL-1(B)	DFF45 - PI3K(PIK3R1)	TNFα - TrkA
CASP3 - IL-1R(1)	DFF45 - PRKACA	TP53 - TRAF2
CASP3 - IRAK2	FADD - IAP(BIRC4)	TP53 - TRAIL-R
CASP3 - NF-κB(NFKB1)	FADD - IL-1(A)	TRAF2 - TRAIL
CASP3 - NF-κB(RELA)	FADD - NGF	TRAIL - TrkA
CASP3 - NGF	FADD - PI3K(PIK3CA)	TRAIL-R - TrkA
CASP3 - PI3K(PIK3CA)	FADD - PRKAR2A	

(e) The true-positive list of Consensus predictions

AKT2 - CASP3	CASP7 - TNF-R1	IAP(BIRC2) - CASP9
AKT2 - IAP(BIRC4)	CASP8 - CASP3	IAP(BIRC2) - IKK
APAF1 - BCL-XL	CASP8 - CASP7	IAP(BIRC3) - CASP7
APAF1 - CASP3	CASP8 - CASP9	IAP(BIRC3) - CASP9
APAF1 - CASP9	CASP8 - FADD	IAP(BIRC3) - TRAF2
BCL-2 - BID	CASP8 - FLIP	IAP(BIRC4) - AKT2
BCL-XL - APAF1	CASP8 - IAP(BIRC4)	IAP(BIRC4) - CASP3
BCL-XL - TP53	CASP8 - TRAF2	IAP(BIRC4) - CASP7
BID - BCL-2	CASP9 - APAF1	IAP(BIRC4) - CASP8
BID - Fas	CASP9 - CASP3	IAP(BIRC4) - CASP9
CASP3 - AKT2	CASP9 - CASP7	IKK - IAP(BIRC2)
CASP3 - APAF1	CASP9 - CASP8	IL-1(B) - IL-1R(1)
CASP3 - CASP8	CASP9 - IAP(BIRC2)	IL-1R(1) - IL-1(B)
CASP3 - CASP9	CASP9 - IAP(BIRC3)	PI3K(PIK3R1) - TrkA
CASP3 - Cn(PPP3CA)	CASP9 - IAP(BIRC4)	TNF-R1 - CASP7
CASP3 - FLIP	Cn(PPP3CA) - CASP3	TP53 - BCL-XL
CASP3 - IAP(BIRC2)	FADD - CASP8	TRAF2 - CASP8
CASP3 - IAP(BIRC4)	FADD - TRAIL	TRAF2 - IAP(BIRC3)
CASP7 - CASP8	FLIP - CASP3	TRAIL - FADD
CASP7 - CASP9	FLIP - CASP8	TRAIL - TRAIL-R
CASP7 - IAP(BIRC2)	Fas - BID	TRAIL-R - TRAIL
CASP7 - IAP(BIRC3)	IAP(BIRC2) - CASP3	TrkA - PI3K(PIK3R1)
CASP7 - IAP(BIRC4)	IAP(BIRC2) - CASP7	

(f) The false-positive list of Consensus predictions

AKT1 - Fas	Cn(PPP3CA) - BCL-XL	NF-κB(NFKB1) - CASP7
AKT2 - IL-3	Cn(PPP3CA) - BID	NF-κB(NFKB1) - IKK
APAF1 - Bax	Cn(PPP3CA) - CASP8	NF-κB(RELA) - CASP7
APAF1 - CASP7	Cn(PPP3CA) - IAP(BIRC2)	NGF - BCL-2
APAF1 - IKK	Cn(PPP3CA) - MyD88	NGF - CASP7
APAF1 - PI3K(PIK3R1)	Cn(PPP3CA) - TP53	NGF - IAP(BIRC3)
APAF1 - TRAF2	Cn(PPP3CA) - TRAF2	PI3K(PIK3CA) - CASP7
BCL-2 - IKK	Cn(PPP3R1) - DFF40	PI3K(PIK3CA) - FADD
BCL-2 - NGF	CytC - CASP7	PI3K(PIK3R1) - APAF1
BCL-2 - PI3K(PIK3R1)	DFF40 - Cn(PPP3R1)	PI3K(PIK3R1) - BCL-2
BCL-XL - Cn(PPP3CA)	DFF40 - MyD88	PI3K(PIK3R1) - CASP7
BCL-XL - IKK	DFF45 - PI3K(PIK3R1)	PI3K(PIK3R1) - CASP8
BCL-XL - TRAF2	FADD - CASP7	PI3K(PIK3R1) - DFF45
BID - CASP7	FADD - PI3K(PIK3CA)	PI3K(PIK3R1) - IKK
BID - Cn(PPP3CA)	Fas - AKT1	PI3K(PIK3R1) - TNF-R1
BID - IKK	Fas - Bax	PI3K(PIK3R1) - TRAF2
Bax - APAF1	Fas - CASP7	PI3K(PIK3R2) - CASP3
Bax - Fas	Fas - TP53	PI3K(PIK3R2) - TNF-R1
Bax - IAP(BIRC4)	IAP(BIRC2) - Cn(PPP3CA)	PI3K(PIK3R2) - TRAF2
Bax - MyD88	IAP(BIRC3) - CASP8	TNF-R1 - IL-3R
CASP3 - CASP7	IAP(BIRC3) - NGF	TNF-R1 - PI3K(PIK3R1)
CASP3 - NF-κB(NFKB1)	IAP(BIRC4) - Bax	TNF-R1 - PI3K(PIK3R2)
CASP3 - PI3K(PIK3R2)	IAP(BIRC4) - MyD88	TNFα - CASP7
CASP3 - TRAF2	IAP(BIRC4) - TRADD	TNFα - CASP9
CASP6 - CASP7	IKK - APAF1	TP53 - Cn(PPP3CA)
CASP6 - IKK	IKK - BCL-2	TP53 - Fas
CASP7 - APAF1	IKK - BCL-XL	TP53 - IKK
CASP7 - BID	IKK - BID	TP53 - TRAF2
CASP7 - CASP3	IKK - CASP6	TP53 - TRAIL-R
CASP7 - CASP6	IKK - MyD88	TRADD - IAP(BIRC4)
CASP7 - CytC	IKK - NF-κB(NFKB1)	TRAF2 - APAF1
CASP7 - FADD	IKK - PI3K(PIK3R1)	TRAF2 - BCL-XL
CASP7 - Fas	IKK - TP53	TRAF2 - CASP3

CASP7 - MyD88	IKK - TRAF2	TRAF2 - CASP7
CASP7 - NF-κB(NFKB1)	IL-3 - AKT2	TRAF2 - Cn(PPP3CA)
CASP7 - NF-κB(RELA)	IL-3 - TRAF2	TRAF2 - IKK
CASP7 - NGF	IL-3R - TNF-R1	TRAF2 - IL-3
CASP7 - PI3K(PIK3CA)	IL-3R - TRAF2	TRAF2 - IL-3R
CASP7 - PI3K(PIK3R1)	IRAK2 - CASP9	TRAF2 - PI3K(PIK3R1)
CASP7 - TNFα	MyD88 - Bax	TRAF2 - PI3K(PIK3R2)
CASP7 - TRAF2	MyD88 - CASP7	TRAF2 - TP53
CASP8 - Cn(PPP3CA)	MyD88 - Cn(PPP3CA)	TRAIL-R - TP53
CASP8 - IAP(BIRC3)	MyD88 - DFF40	TRAIL-R - TrkA
CASP8 - PI3K(PIK3R1)	MyD88 - IAP(BIRC4)	TrkA - TRAIL-R
CASP9 - IRAK2	MyD88 - IKK	
CASP9 - TNFα	NF-κB(NFKB1) - CASP3	

Note : The abbreviations used are: AIF, apoptosis-inducing factor, mitochondrion-associated, 1 (AIFM1); AKT1, RAC-alpha serine/threonine-protein kinase; AKT2, RAC-beta serine/threonine-protein kinase; AKT3, RAC-gamma serine/threonine-protein kinase; APAF1, apoptotic peptidase activating factor 1; BCL-2, B-cell lymphoma 2; BCL-XL, BCL extra-large; BID, BH3 interacting domain death agonist; Bax, BCL-2-associated X protein; CASP3/6/7/8/9, caspase-3/6/7/8/9; Cn(CHP), calcineurin B homologous protein 1; Cn(CHP2), calcineurin B homologous protein 2; Cn(PPP3CA), protein phosphatase 3 catalytic subunit alpha isoform; Cn(PPP3R1), protein phosphatase 3 regulatory subunit 1; CytC, cytochrome C; DFF40, DNA fragmentation factor, 40kDa, beta polypeptide; DFF45, DNA fragmentation factor, 45kDa, alpha polypeptide; FADD, Fas-associated via death domain; FLIP, FLICE/CASP8 inhibitory protein (CASP8 and FADD-like apoptosis regulator, CFLAR); Fas, tumor necrosis factor receptor (TNF) superfamily member 6; IAP, inhibitor of apoptosis; BIRC2/3/4, baculoviral IAP repeat-containing protein 2/3/4; IκBα, nuclear factor of kappa light polypeptide gene enhancer in B-cells inhibitor alpha; IKK, inhibitor of nuclear factor kappa-B kinase; IL-1(A), interleukin-1 alpha; IL-1(B), interleukin-1 beta; IL-1R(1), type 1 interleukin-1 receptor; IL-1R(RAP), interleukin-1 receptor accessory protein; IL-3, interleukin-3; IL-3R, interleukin-3 receptor; IRAK2/4, interleukin-1 receptor-associated kinase 2/4; MyD88, myeloid differentiation primary response protein MyD88; NF-κB(NFKB1), nuclear factor of kappa light polypeptide gene enhancer in B-cells; NF-κB(RELA), nuclear factor of kappa light polypeptide gene enhancer in B-cells 3; NGF, nerve growth factor (beta polypeptide); PI3K, phosphatidylinositide 3-kinase; PIK3CA, PI3K subunit alpha; PIK3CG, PI3K subunit gamma; PIK3R1, PI3K regulatory subunit alpha; PIK3R2, PI3K regulatory subunit beta; PRKACA, cyclic adenosine monophosphate (cAMP)-dependent protein kinase catalytic subunit alpha; PRKAR2A, cAMP-dependent protein kinase type II-alpha regulatory subunit; TNFα, tumor necrosis factor; TNF-R1, TNF receptor superfamily member 1A; TP53, cellular tumor antigen p53; TRADD, TNF receptor type 1-associated death domain protein; TRAF2, TNF receptor-associated factor 2; TRAIL, TNF receptor superfamily member 10; TRAIL-R, TNF receptor superfamily member 10B; TrkA, neurotrophic tyrosine kinase receptor type 1.
